# Fracture Resistance of Artificially Aged Three-Unit Interim Fixed Dental Prostheses: A Comparison of an Additively Manufactured Photopolymer, a Milled PMMA, and a Conventionally Autopolymerized PEMA-Based Resin

**DOI:** 10.3390/polym18172140

**Published:** 2026-09-02

**Authors:** Özlem Özişçi, Elif Irmak Gökmen

**Affiliations:** Department of Prosthodontics, Faculty of Dentistry, Süleyman Demirel University, Çünür, 32260 Isparta, Türkiye; elifirmakgokmen.97@gmail.com

**Keywords:** interim fixed dental prosthesis, PMMA, PEMA, photopolymer resin, vat photopolymerization, CAD/CAM, thermocycling, fracture resistance

## Abstract

Interim fixed dental prostheses (FDPs) must withstand functional loading, thermal fluctuation, and moisture, yet comparative fracture resistance of additively manufactured, CAD/CAM-milled, and conventionally polymerized provisional materials after artificial aging remains unclear. Ninety three-unit interim FDPs (*n* = 30 per material) were fabricated from an additively manufactured photopolymer resin, CAD/CAM-milled poly(methyl methacrylate) (PMMA), or a conventionally autopolymerized powder–liquid poly(ethyl methacrylate) (PEMA)-based resin, and divided into non-aged and aged subgroups (5000 thermal cycles, 5/55 °C; *n* = 15). Restorations were seated without cementation on rigid titanium dies and loaded at the pontic to fracture (4 mm spherical indenter, 0.5 mm/min). The material-fabrication workflow grouping showed the largest effect on maximum fracture load (*p* < 0.001, η^2^p = 0.923): CAD/CAM-milled PMMA (1131.95 ± 135.31 N) > PEMA-based resin (475.06 ± 171.96 N) > photopolymer resin (163.34 ± 36.79 N). A material × aging interaction (*p* = 0.009) indicated that thermocycling reduced fracture load only in milled PMMA. For time to maximum load, only the interaction was significant (*p* = 0.008), driven by the photopolymer group. Fracture location and pattern varied by group. Within the limitations of this in vitro study, differences in maximum fracture load were primarily associated with the investigated material-fabrication workflow combinations, whereas aging effects were material-dependent.

## 1. Introduction

Interim fixed dental prostheses (FDPs) constitute an essential component of contemporary fixed prosthodontic treatment by protecting prepared abutments, preserving occlusal stability, maintaining periodontal health, and restoring function and esthetics until delivery of the definitive prosthesis. In addition to serving as biological and mechanical protective restorations, interim FDPs enable clinicians to evaluate occlusal relationships, phonetics, and esthetic outcomes before definitive treatment [1]. Although intended for temporary use, interim restorations frequently remain in service for extended periods because of implant osseointegration, multidisciplinary treatment sequencing, or laboratory turnaround time, with a reported mean functional service period of approximately 44.5 days and a fracture incidence approaching 13% during this interval [2]. Consequently, interim restorations are continuously subjected to cyclic occlusal loading, thermal fluctuations, and moisture, making adequate mechanical performance, particularly fracture resistance, essential for reliable clinical function [2,3].

Resin-based provisional materials remain the materials of choice for interim restorations because of their favorable esthetics, ease of manipulation, repairability, polishability, and cost-effectiveness [4]. However, their mechanical performance is strongly influenced not only by the intrinsic properties of the material but also by its manufacturing route, polymer chemistry, monomer–polymer composition, degree of conversion, and processing conditions [5,6]. Conventionally polymerized powder–liquid methacrylate-based resins, industrially prepolymerized CAD/CAM-milled polymethyl methacrylate (PMMA), and additively manufactured photopolymer resins are produced through fundamentally different polymerization pathways that generate distinct polymer-network architectures [5]. These differences may influence residual monomer content, cross-link density, molecular homogeneity, internal porosity, and polymer chain organization, which in turn may affect crack initiation, crack propagation, and ultimately fracture resistance [2,5]. Studies of light-polymerized dental resin systems have demonstrated that variations in material composition and degree of conversion may influence mechanical properties, further emphasizing the importance of polymer-network characteristics when interpreting the mechanical behavior of resin-based dental materials [7]. Accordingly, material chemistry and fabrication workflow should be considered jointly when interpreting the mechanical behavior of resin-based provisional restorations.

The rapid integration of digital manufacturing technologies has substantially transformed the fabrication of interim prostheses [8]. CAD/CAM milling utilizes industrially prepolymerized PMMA discs processed under precisely controlled temperature and pressure conditions, producing highly homogeneous materials characterized by minimal polymerization shrinkage, reduced internal porosity, low residual monomer content, and excellent dimensional stability [9,10]. CAD/CAM-milled PMMA has consistently demonstrated superior fracture resistance, flexural strength, marginal accuracy, and long-term mechanical reliability compared with conventionally fabricated provisional restorations [11,12].

Conversely, additive manufacturing has emerged as an attractive digital alternative because of its ability to fabricate geometrically complex restorations with minimal material waste, reduced production time, and excellent manufacturing reproducibility [2,11]. Unlike subtractive manufacturing, however, additively manufactured photopolymer resins are fabricated through a layer-by-layer polymerization process in which interlayer adhesion, build orientation, layer thickness, printing parameters, and post-curing conditions substantially influence the resulting mechanical properties [13,14]. The fracture behavior of printed provisional restorations remains considerably more variable than that of industrially milled materials, and conflicting findings persist regarding their fracture resistance, wear behavior, dimensional stability, and sensitivity to post-processing procedures [15,16]. Direct comparative studies of interim FDPs have further shown that the mechanical performance of additively manufactured restorations may vary according to the specific resin system, printing technology, and processing conditions employed [17]. Similar differences have also been reported for the optical, physical, and surface properties of provisional restorative materials fabricated using CAD/CAM milling and additive manufacturing, further emphasizing the influence of manufacturing technology on the overall structural performance of interim restorations [18].

Beyond manufacturing technology, the long-term clinical performance of provisional restorations is considerably influenced by hydrothermal aging. Water sorption promotes polymer plasticization and hydrolytic degradation, reducing intermolecular interactions and facilitating crack initiation and propagation under functional loading. Simultaneously, repeated thermal cycling generates internal stresses through cyclic expansion and contraction, thereby accelerating fatigue damage within the polymer network [18,19]. Although numerous investigations have demonstrated that artificial aging adversely affects the mechanical performance of provisional restorative materials, the magnitude of degradation appears to depend on both material composition and manufacturing technique [2,11]. Industrially polymerized CAD/CAM PMMA generally exhibits superior baseline mechanical properties, whereas conventionally polymerized powder–liquid methacrylate resins may undergo continued post-curing reactions that partially compensate for aging-related degradation. Likewise, the aging behavior of additively manufactured photopolymer resins is strongly influenced by printing parameters, degree of conversion, and post-curing efficiency [13,16]. Recent investigations have further demonstrated that hydrothermal aging can produce material-dependent changes in the mechanical properties of additively manufactured interim resins, with the magnitude of degradation varying considerably among resin formulations and processing conditions [20,21,22]. Consequently, the interaction between manufacturing technology and artificial aging remains insufficiently understood.

Another factor influencing fracture behavior is the supporting substrate used during mechanical testing. Previous investigations have employed natural teeth, resin dies, or models incorporating simulated periodontal ligament compliance, whereas rigid testing substrates have also been used to improve experimental standardization. The mechanical properties and compliance of the supporting substrate may influence load transfer and stress distribution within an FDP. In the present study, a standardized, non-compliant titanium die model was therefore used to minimize substrate-related variability and provide identical support conditions for all specimens. Previous studies have compared the mechanical performance of interim FDPs fabricated using conventional, subtractive, and additive manufacturing approaches [2,10,11]. However, direct comparison across studies remains difficult because of differences in material composition, polymerization mechanisms, prosthesis design, testing protocols, and aging conditions. Nevertheless, the fracture behavior of different material-fabrication workflow combinations following hydrothermal aging under identical geometric and rigid-support conditions remains insufficiently characterized. Further controlled comparisons under standardized aging and mechanical testing conditions are therefore warranted.

Therefore, the aim of the present in vitro study was to compare the fracture resistance of three-unit interim fixed dental prostheses fabricated from an additively manufactured photopolymer resin, CAD/CAM-milled PMMA, and a conventionally polymerized powder–liquid methacrylate-based resin following artificial aging using a standardized rigid titanium die model. The null hypotheses were that (1) restorative material would not influence fracture resistance, (2) artificial aging would not affect fracture resistance, and (3) no interaction would exist between restorative material and aging condition.

## 2. Materials and Methods

### 2.1. Study Design

This in vitro study evaluated the influence of fabrication material and artificial aging on the fracture behavior of three-unit interim fixed dental prostheses (FDPs). A total of 90 restorations were fabricated using three commercially available resin-based provisional restorative materials, each representing a distinct fabrication workflow: an additively manufactured photopolymer resin (Cyclone Temp Premium Resin, shade A2; ON DENT, İzmir, Türkiye), an industrially prepolymerized CAD/CAM-milled polymethyl methacrylate (PMMA) disc (Alliance Disc, shade A2; Turkuaz Dental, İzmir, Türkiye), and a conventionally polymerized powder–liquid methacrylate-based resin (Dentalon^®^ Plus, shade A2; Kulzer GmbH, Hanau, Germany). Thirty restorations were fabricated from each material and equally allocated to non-aged and artificially aged subgroups (*n* = 15), resulting in six experimental groups (Table 1).

All specimens represented a standardized mandibular three-unit FDP replacing the mandibular first molar (FDI tooth #36), with the mandibular second premolar (FDI tooth #35) and second molar (FDI tooth #37) serving as abutments. Prosthesis contour, connector dimensions, pontic morphology, occlusal anatomy, marginal configuration, and cement space were standardized using a single digital design to ensure that only the fabrication material and artificial aging condition differed among the experimental groups.

The primary outcome variable was maximum fracture load (N). Secondary outcome variables included time to maximum load (s), fracture location, and fracture pattern. All specimen fabrication, artificial aging procedures, and mechanical testing were performed by a single calibrated operator under standardized laboratory conditions to minimize procedural variability.

### 2.2. Master Model Preparation and Fabrication of the Titanium Testing Model

A mandibular dentoform model with anatomically contoured resin teeth (Frasaco GmbH, Tettnang, Germany) served as the basis for preparation of the experimental abutments. The mandibular first molar (FDI tooth #36) was removed to simulate a posterior edentulous space, whereas the mandibular second premolar (FDI tooth #35) and second molar (FDI tooth #37) were prepared as abutments for a three-unit FDP. Tooth preparations were completed using a circumferential shoulder finish line with rounded internal line angles employing calibrated diamond rotary instruments. Preparation geometry, total occlusal convergence, and inter-abutment parallelism were continuously verified using a dental surveyor to obtain a standardized path of insertion for all restorations.

**Figure 1:**
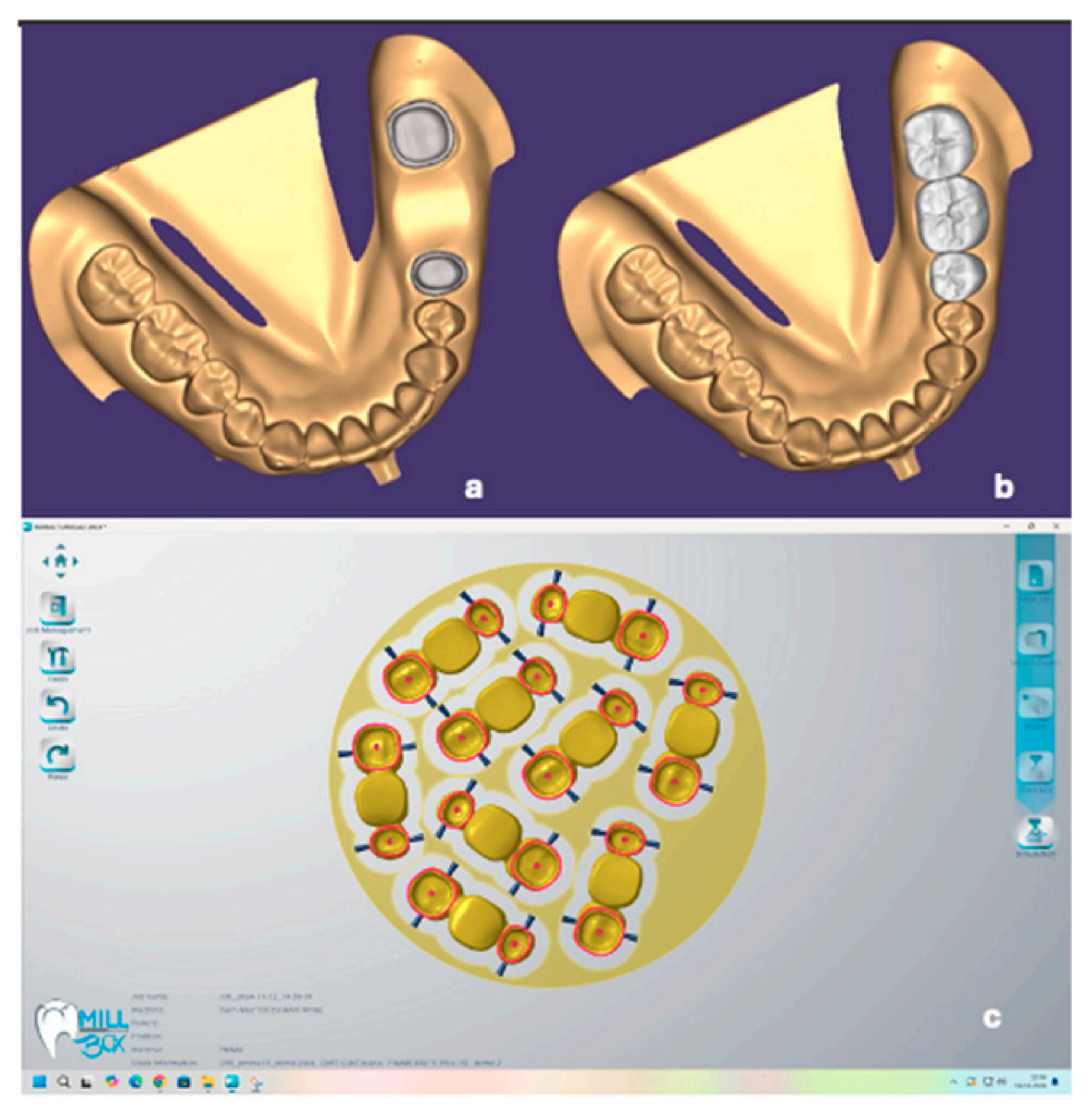
Digital design and premanufacturing setup of the standardized three-unit interim fixed dental prosthesis. (**a**) Laboratory scanning of the prepared abutment model; (**b**) completed virtual design of the three-unit interim FDP on the digital model; and (**c**) representative digital positioning of the standardized restoration design before fabrication.

Following preparation, the dentoform was digitized using a laboratory scanner (Dental Wings Series 7, Dental Wings Inc., Montreal, QC, Canada). The resulting stereolithography (STL) dataset was imported into exocad DentalCAD software 3.3 Chemnit (exocad GmbH, Darmstadt, Germany), where a standardized three-unit interim FDP was designed. Prosthesis contour, marginal configuration, connector dimensions, pontic morphology, occlusal anatomy, and cement space were standardized digitally to eliminate geometric variability among specimens (Figure 1).

To provide a standardized, rigid, and reproducible testing substrate, titanium replicas of the prepared mandibular second premolar and second molar were manufactured directly from the STL dataset using CAD/CAM machining and rigidly fixed within a custom-fabricated stainless-steel base. The resulting non-compliant die assembly reproduced the standardized inter-abutment relationship while eliminating variability associated with natural teeth, resin dies, and simulated periodontal ligament materials, thereby providing identical support conditions for all fracture tests.

Connector cross-sectional areas were standardized at 10 mm^2^ for the mesial connector and 12 mm^2^ for the distal connector, consistent with previously reported dimensions for mechanically tested three-unit interim FDPs. The pontic was designed with a modified ridge-lap configuration. Because all restorations originated from the same digital design, prosthesis geometry remained identical irrespective of the subsequent fabrication workflow.

No soft-tissue analogue or simulated mucosal material was incorporated beneath the pontic. Consequently, the pontic remained completely unsupported throughout mechanical testing, with no contact between its tissue surface and the underlying testing assembly. This configuration allowed the applied load to be transmitted exclusively through the retainer–abutment complex.

The identical STL dataset was used directly for fabrication of both the additively manufactured photopolymer resin restorations and the CAD/CAM-milled PMMA restorations. In contrast, restorations fabricated from the conventionally polymerized methacrylate-based resin were produced indirectly using a silicone-index duplication workflow. A reference restoration fabricated from the identical STL dataset served as the master pattern for producing a two-piece polyvinyl siloxane (PVS) duplication index, thereby transferring the standardized digital design to the conventional fabrication procedure. Consequently, the same digital design was used for all specimens to standardize prosthesis geometry, including contour, connector dimensions, pontic morphology, occlusal anatomy, marginal configuration, and cement space. This standardized workflow ensured that differences observed during mechanical testing reflected the combined influence of material characteristics and fabrication workflow rather than geometric variability among restorations.

### 2.3. Fabrication of Interim Fixed Dental Prostheses

A total of 90 interim FDPs were fabricated using three commercially available resin-based provisional restorative materials, each representing a distinct fabrication workflow: an additively manufactured photopolymer resin, an industrially prepolymerized CAD/CAM-milled PMMA, and a conventionally polymerized powder–liquid methacrylate-based resin. All restorations were fabricated according to the standardized design and duplication procedures described above.

#### 2.3.1. Additively Manufactured Photopolymer Resin

Interim FDPs were fabricated from a light-curable photopolymer resin developed for interim crown and bridge restorations (Cyclone Temp Premium Resin, shade A2; ON DENT, İzmir, Türkiye) using a low-force stereolithography (LFS) three-dimensional printer (Form 3B; Formlabs Inc., Somerville, MA, USA). The Form 3B was selected because it was the LFS printing platform available in our laboratory and permitted the processing of third-party photopolymer resins through Open Material Mode. The printer employs a 405-nm violet laser; however, Cyclone Temp Premium Resin was not included among the resin–printer combinations validated by the printer manufacturer for this platform. Consequently, fabrication was performed using Open Material Mode. No manufacturer-validated Form 3B exposure profile was available for this resin, and therefore a printer-specific manufacturer-recommended exposure condition could not be tested. In addition, the resin-specific photoinitiator absorption spectrum, critical exposure, penetration depth, and optimal energy dose were not independently characterized. Accordingly, the processing parameters used in Open Material Mode should be regarded as study-specific rather than as manufacturer-optimized conditions for the Cyclone Temp-Form 3B combination.

The standardized STL dataset was imported into PreForm software 3.62.1 (Formlabs Inc.). All specimens were printed using the same Open Material Mode exposure profile at a layer thickness of 100 µm, with identical build orientation and support configuration. Following printing, the restorations were washed twice in 99% isopropyl alcohol using a Form Wash unit (Formlabs Inc.) to remove uncured resin. After complete evaporation of residual alcohol, all support structures were carefully removed.

Post-curing was performed in a Form Cure unit (Formlabs Inc.) according to the resin manufacturer’s instructions for use. Post-polymerization was carried out under ambient atmospheric conditions without inert-gas protection. After post-curing, the restorations were finished using fine-grit tungsten carbide rotary instruments and polished with a standardized silicone polishing system. To minimize manufacturing-related variability, all specimens were fabricated using identical printing parameters, post-processing procedures, and finishing protocols.

#### 2.3.2. Conventionally Polymerized Methacrylate-Based Resin

Interim FDPs were fabricated from a self-curing powder–liquid methacrylate-based provisional restorative resin (Dentalon^®^ Plus, shade A2; Kulzer GmbH, Hanau, Germany) using a conventional silicone-index duplication technique.

According to the manufacturer, the powder component consists primarily of polyethyl methacrylate (PEMA) and polymethyl acrylate (PMA), whereas the liquid component contains n-butyl methacrylate, ethyl methacrylate, and urethane acrylate.

To ensure complete geometric standardization, a reference restoration fabricated from the identical STL dataset served as the master pattern for producing a two-piece polyvinyl siloxane (PVS) duplication index (Presigum Putty and Light Body; President Dental GmbH, Allershausen, Germany). The duplication index incorporated complementary key-and-keyway features to ensure reproducible repositioning and served as the template for reproducing identical prosthesis contour, connector dimensions, pontic morphology, occlusal anatomy, and marginal configuration.

The powder and liquid components were proportioned and mixed according to the manufacturer’s instructions. The mixed material was introduced into the closed PVS duplication index during its working phase and allowed to polymerize undisturbed at room temperature through chemical autopolymerization. After complete polymerization, the restorations were removed from the mold, finished using tungsten carbide rotary instruments (Komet-Brasseler GmbH, Lemgo, Germany), and polished according to the identical protocol applied to the digitally fabricated restorations.

#### 2.3.3. CAD/CAM-Milled PMMA

Interim FDPs were fabricated from industrially prepolymerized PMMA discs (Alliance Disc, shade A2; Turkuaz Dental, İzmir, Türkiye) using a five-axis CAD/CAM milling unit (YenaDent; Yena Makine, İzmir, Türkiye).

The identical STL dataset used for the additively manufactured restorations was employed for milling to ensure complete geometric equivalence among all specimens.

Industrial prepolymerization of CAD/CAM PMMA materials under controlled temperature and pressure has generally been associated with high structural homogeneity, reduced internal porosity, and lower residual monomer content compared with conventionally polymerized materials. These characteristics were not independently quantified for the material investigated in the present study. Following milling, the restorations were separated from the supporting sprues, finished using fine-grit tungsten carbide rotary instruments, and polished according to the identical finishing protocol applied to the other two fabrication groups. No additional post-polymerization procedure was performed.

#### 2.3.4. Quality Control

Following fabrication, all restorations were visually inspected under 2.5× magnification to identify surface defects, porosity, incomplete polymerization, marginal discrepancies, or visible cracks. Specimens presenting any detectable defects were discarded and remanufactured before the aging procedures.

Each restoration was seated on the standardized titanium testing model to verify complete seating and passive fit. Marginal adaptation was visually confirmed before fracture testing. No luting agent was used during specimen seating or fracture testing.

### 2.4. Artificial Aging Protocol

Following fabrication, specimens assigned to the artificially aged subgroups (*n* = 15 per fabrication technique; N = 45 total) were subjected to thermocycling using a dedicated thermocycling device (SD Mechatronik GmbH, Feldkirchen-Westerham, Germany). A total of 5000 thermal cycles were performed between 5 °C and 55 °C, with a dwell time of 25 s in each bath and a transfer time of 10 s. Thermocycling was selected as a standardized hydrothermal-aging procedure to isolate the effects of repeated temperature fluctuation and water exposure without introducing mechanical fatigue as an additional degradation factor. The use of 5000 cycles between 5 °C and 55 °C was based on previously reported thermocycling protocols for dental restorative and interim FDP materials [14,20,23]. Distilled water was refreshed periodically throughout the thermocycling procedure to maintain consistent thermal conditions and minimize contamination.

Non-aged specimens (*n* = 15 per fabrication technique; N = 45 total) were stored in distilled water at 37 °C for 24 h prior to fracture testing, thereby standardizing baseline hydration across groups without additional thermal or mechanical fatigue.

### 2.5. Fracture Load Testing

Fracture resistance testing was performed using a universal testing machine (AG-X Plus, 100 kN load cell; Shimadzu Corporation, Kyoto, Japan). Before testing, each restoration was seated on the standardized rigid titanium die assembly without cementation and stabilized within a custom-fabricated stainless-steel fixture to ensure reproducible positioning throughout the experiment. No luting agent was used so that the restoration–die interface conditions remained identical across all experimental groups and cement-dependent variability did not confound between-group comparisons. This configuration was selected for experimental standardization; however, it does not reproduce the clinical tooth–cement–restoration complex and therefore limits direct extrapolation of the measured fracture loads to clinical conditions.

A compressive load was applied perpendicular (90°) to the occlusal surface of the pontic (FDI tooth #36) at the central fossa using a 4-mm-diameter stainless-steel spherical indenter. The selected loading configuration provided standardized point-contact loading while minimizing localized contact stress concentrations and is consistent with loading protocols commonly reported for fracture testing of interim fixed dental prostheses. Load was applied at a constant crosshead speed of 0.5 mm/min until structural failure was detected and the maximum fracture load was recorded.

### 2.6. Statistical Analysis

Statistical analyses were performed using Minitab Statistical Software (Version 17; Minitab LLC, State College, PA, USA) and jamovi (Version 2.3.28; The jamovi Project, Sydney, Australia). The normality of continuous variables was assessed using the Shapiro–Wilk test, whereas homogeneity of variances was evaluated using Levene’s test. A two-sided significance level of α = 0.05 was adopted for all statistical analyses.

Maximum fracture load demonstrated a normal distribution and was therefore analyzed using a two-way analysis of variance (ANOVA) with restorative material (additively manufactured photopolymer resin, CAD/CAM-milled PMMA, and conventionally polymerized methacrylate-based resin) and aging condition (non-aged and artificially aged) as fixed factors, together with their interaction term. When statistically significant differences were detected, pairwise comparisons were performed using the Bonferroni adjustment.

Variables that violated the assumptions of normality (time to maximum load) were analyzed using a two-way robust ANOVA, followed by MCP2A robust multiple-comparison testing for pairwise group comparisons. Robust ANOVA was selected because it provides reliable inference in factorial designs when normality assumptions are not satisfied.

For categorical variables (fracture location and fracture pattern), results are presented descriptively as frequencies and percentages.

Continuous variables satisfying the assumptions of normality are reported as mean ± standard deviation (SD), whereas non-normally distributed variables are presented as median (minimum–maximum). Effect sizes for all factorial analyses are reported as partial eta squared (η^2^p) together with their corresponding 95% confidence intervals (95% CI).

## 3. Results

A total of 90 interim fixed dental prostheses were included in the study, comprising 30 restorations for each restorative material, equally distributed between the non-aged and artificially aged subgroups (*n* = 15). All specimens completed the experimental protocol and were included in the statistical analysis; therefore, no specimens were excluded.

### 3.1. Maximum Fracture Load

**Figure 2:**
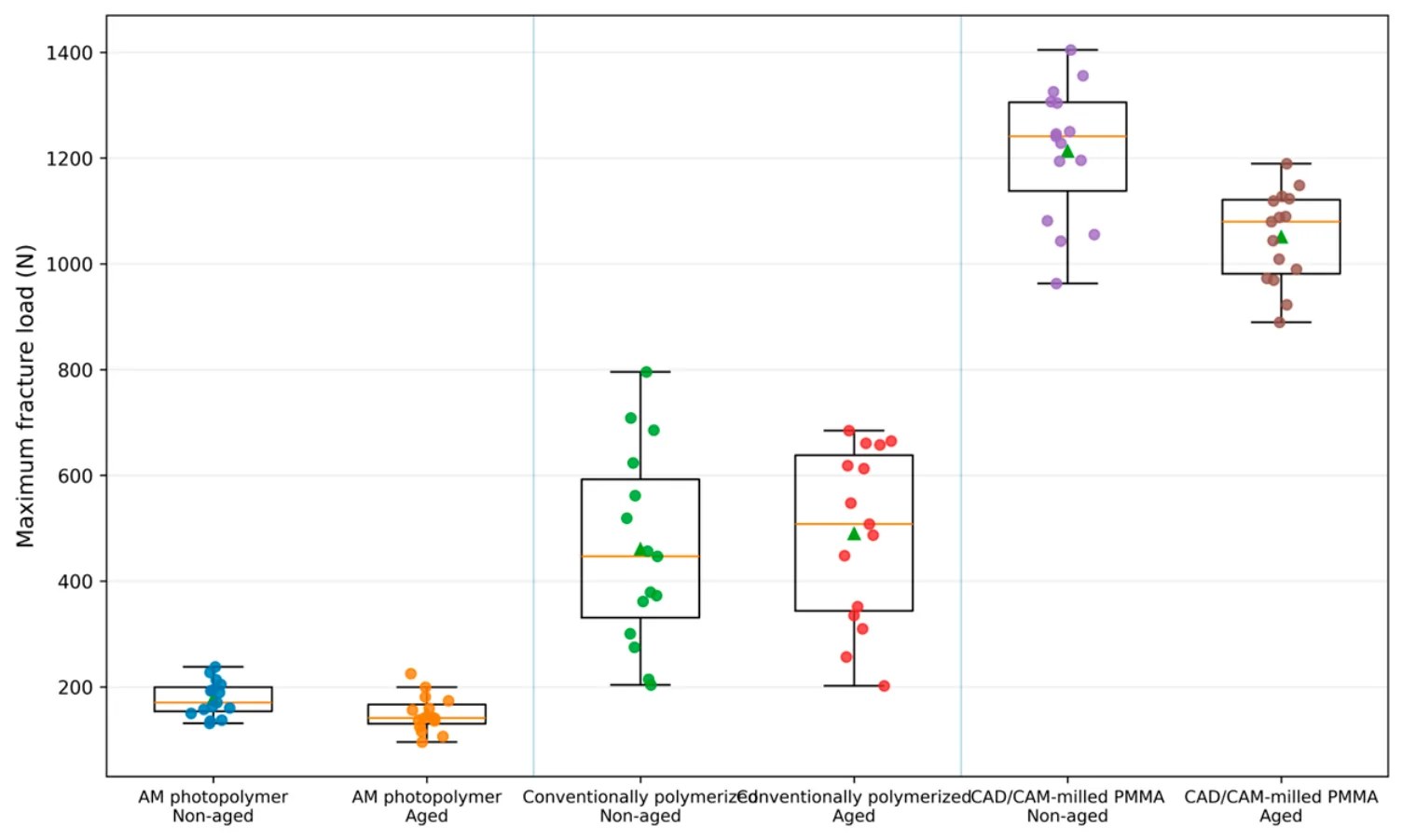
Distribution of maximum fracture load according to restorative material and aging condition. Individual observations (*n* = 15 per group) are shown together with box-and-whisker plots and group means. AM, additively manufactured; PMMA, poly(methyl methacrylate).

Maximum fracture load differed significantly according to restorative material and artificial aging (Table 2). Two-way ANOVA demonstrated statistically significant main effects of restorative material (F = 505.985, *p* < 0.001, partial η^2^ = 0.923), artificial aging (F = 4.499, *p* = 0.037, partial η^2^ = 0.051), and their interaction (F = 4.997, *p* = 0.009, partial η^2^ = 0.106). The group-wise distribution of maximum fracture load values is illustrated in Figure 2, allowing direct visualization of both between-material differences and aging-related changes.

Across both aging conditions, the CAD/CAM-milled PMMA group demonstrated the highest maximum fracture load (1131.95 ± 135.31 N), followed by the conventionally polymerized methacrylate-based resin (Dentalon^®^ Plus; 475.06 ± 171.96 N) and the additively manufactured photopolymer resin (Cyclone Temp Premium Resin; 163.34 ± 36.79 N). Bonferroni-adjusted pairwise comparisons demonstrated statistically significant differences among all restorative materials (*p* < 0.05).

Artificial aging produced a significant reduction in maximum fracture load overall. However, post hoc comparisons demonstrated that this reduction occurred exclusively in the CAD/CAM-milled PMMA group (1213.06 ± 126.65 N vs. 1050.83 ± 88.22 N), whereas no statistically significant aging-related differences were identified for the additively manufactured photopolymer resin or Dentalon^®^ Plus (*p* > 0.05).

### 3.2. Time to Maximum Load

Time to maximum load did not differ significantly according to restorative material or artificial aging; however, a significant material × aging interaction was observed (Table 3). Two-way robust ANOVA revealed no significant main effects of restorative material (*p* = 0.743) or artificial aging (*p* = 0.699). In contrast, a statistically significant interaction between restorative material and aging condition was observed (F = 9.775, *p* = 0.008, partial η^2^ = 0.189).

Robust multiple comparisons demonstrated that this interaction was attributable to the additively manufactured photopolymer resin, in which artificial aging significantly reduced the time to maximum load (281.92 s vs. 233.01 s). No significant aging-related differences were identified for Dentalon^®^ Plus or the CAD/CAM-milled PMMA group.

### 3.3. Fracture Location

Fracture location exhibited distinct distribution patterns across restorative materials and aging conditions (Table 4). Fractures in the additively manufactured photopolymer resin group occurred predominantly at the mesial connector under both aging conditions. In the Dentalon^®^ Plus group, the predominant fracture location shifted from the mesial connector in non-aged specimens to the distal connector following artificial aging. In contrast, fractures in the CAD/CAM-milled PMMA group occurred predominantly at the distal connector, irrespective of aging condition.

Pontic fractures were observed exclusively in the additively manufactured photopolymer resin group, whereas simultaneous fractures involving both connectors were identified only in the Dentalon^®^ Plus and CAD/CAM-milled PMMA groups.

### 3.4. Fracture Pattern

**Figure 3:**
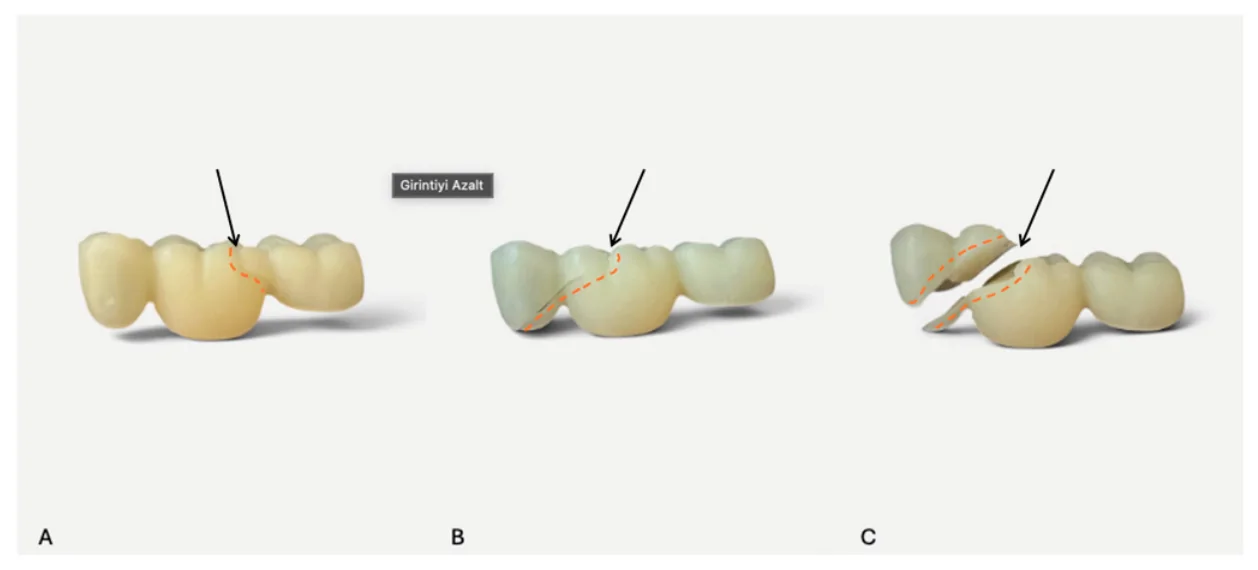
Representative fracture patterns of the three-unit interim fixed dental prostheses after fracture testing. White arrows indicate the principal fracture line or connector region involved in failure. (**A**) Partial fracture: a visible crack was present within the restoration without complete separation of the fractured components, and the prosthesis maintained its overall structural integrity. (**B**) Incomplete fracture: partial structural separation involving the connector region was present; however, the fractured components remained connected and the prosthesis retained its overall integrity. (**C**) Complete fracture: catastrophic failure with complete separation of the restoration into two or more independent fragments; the fracture line traversed the full cross-section of the connector.

Fracture patterns varied across restorative materials and aging conditions, with incomplete fracture being the predominant failure pattern overall (Table 5; Figure 3). The additively manufactured photopolymer resin exhibited exclusively partial and incomplete fractures, with no complete fractures recorded in either aging condition.

In the Dentalon^®^ Plus group, complete fractures occurred primarily in non-aged specimens, whereas artificial aging was associated with an increased frequency of incomplete fractures. The CAD/CAM-milled PMMA group also demonstrated predominantly incomplete fractures under both aging conditions, while complete fractures were observed only occasionally.

## 4. Discussion

The present study demonstrated that the investigated material-fabrication workflow grouping showed a substantially larger statistical effect on maximum fracture load than artificial aging. A significant material × aging interaction further indicated that the effect of thermocycling was material-dependent rather than uniform across the investigated restorations. Accordingly, the first and second null hypotheses were rejected, and the significant interaction led to rejection of the third null hypothesis. Because fracture testing was performed under standardized, non-cemented, rigid-die conditions without periodontal ligament simulation, the absolute fracture load values reported here should be interpreted as comparative laboratory indicators of relative performance rather than as direct predictors of clinical failure thresholds.

Among the materials investigated, CAD/CAM-milled PMMA exhibited the highest fracture resistance, followed by the conventionally polymerized methacrylate-based resin and the additively manufactured photopolymer resin. This ranking is consistent with previous studies reporting favorable mechanical performance for industrially prepolymerized PMMA [2,10]. Because each fabrication workflow was represented by a single commercially available material, material composition and manufacturing technique were not independently varied within a full factorial design. Accordingly, the observed differences should be interpreted as the performance of the three specific material-fabrication workflow combinations investigated rather than as evidence of an inherent advantage of one manufacturing technology over another [5,13,24]. Because degree of conversion, residual monomer content, water sorption/solubility, porosity, and interlayer integrity were not directly measured, the mechanistic interpretations discussed below should be regarded as hypothesis-generating explanations based on previous literature rather than as experimentally verified mechanisms in the materials tested here.

The superior performance of the CAD/CAM-milled PMMA material is consistent with previous laboratory investigations demonstrating that industrially polymerized PMMA exhibits greater fracture resistance, flexural strength, dimensional stability, and structural homogeneity than conventionally fabricated provisional materials [9,12,25]. Industrial prepolymerization under controlled temperature and pressure has been associated in previous studies with relatively high conversion, reduced residual monomer content, and greater structural homogeneity [9]. These characteristics may reduce potential stress-concentration sites and improve resistance to crack propagation, and therefore may have contributed to the greater fracture loads observed for the CAD/CAM-milled PMMA group [2,10]. However, these physicochemical characteristics were not directly quantified in the material investigated in the present study.

The present findings are also in agreement with those of Henderson et al., who reported significantly higher failure loads for CAD/CAM-milled interim fixed dental prostheses than for conventionally fabricated and three-dimensionally printed restorations [2]. Differences in resin formulation, photoinitiator chemistry, filler composition, degree of conversion, and post-curing protocol may have contributed to differences in absolute fracture resistance between the studies [14,15]. Similar considerations also apply when comparing the present findings with those of Reymus et al. and Greuling et al., who investigated different commercially available photopolymer systems produced using alternative printing technologies and post-processing protocols [11,14]. Therefore, comparisons among studies should primarily be interpreted in terms of general mechanical trends rather than absolute fracture load values.

Dentalon^®^ Plus exhibited intermediate fracture resistance, with values significantly lower than those of CAD/CAM-milled PMMA but higher than those of the additively manufactured photopolymer resin. Unlike industrially polymerized CAD/CAM materials, conventionally polymerized powder–liquid methacrylate-based resins are more susceptible to operator-dependent processing, polymerization shrinkage, and residual monomer formation, which may adversely affect their mechanical properties [19,24]. The comparatively greater fracture resistance of Dentalon^®^ Plus relative to the investigated photopolymer may be consistent with differences in polymerization and structural organization between conventionally polymerized and layer-by-layer fabricated materials. However, polymer-network characteristics and residual monomer content were not directly characterized in the present study.

The additively manufactured photopolymer resin (Cyclone Temp Premium Resin) exhibited the lowest fracture resistance among the investigated materials. The mechanical performance of printed interim restorative materials is influenced by resin formulation, build orientation, layer thickness, interlayer adhesion, and post-curing conditions [13,14]. Previous studies have suggested that layer-by-layer fabrication may introduce interlayer regions that can influence fracture behavior when interlayer bonding is weaker than the bulk material [13,16]. These material and processing related factors may have contributed to the comparatively low fracture loads observed in the present study [25]. However, no fractographic or microstructural analysis was performed in the present study, and the location of crack initiation relative to layer interfaces therefore could not be established.

Beyond these general considerations, the absolute magnitude of the fracture loads recorded for the additively manufactured group warrants cautious interpretation. The mean fracture load of 163.34 ± 36.79 N was markedly lower than values reported for several previously investigated additively manufactured interim FDP systems [11,14]. One possible explanation is the specific resin–printer–post-curing combination used in the present study. The term “resin-printer incompatibility” in this context should not be interpreted as evidence of a simple wavelength mismatch. The Form 3B uses a 405-nm violet laser; however, successful photopolymerization depends not only on nominal wavelength but also on the overlap between the emission spectrum and photoinitiator absorption, as well as the resin-specific critical exposure, penetration depth, scanning strategy, and delivered energy dose. Because these parameters were not characterized for the Cyclone Temp-Form 3B combination, adequate resin-specific exposure could not be confirmed. Open Material Mode was required because the investigated third-party resin was not validated for this printer platform. Accordingly, suboptimal exposure remains a plausible explanation for the low fracture loads, but it was not experimentally demonstrated in the present study. However, degree of conversion, interlayer bond quality, and microstructural characteristics were not directly evaluated. Therefore, these mechanisms should be regarded as plausible explanations rather than experimentally verified causes of the comparatively low fracture resistance. The present findings should consequently be interpreted as specific to the resin–printer–post-curing combination investigated rather than as representative of additive manufacturing as a fabrication technology.

Artificial aging exerted a statistically significant but comparatively modest effect on fracture resistance. More importantly, the significant material × aging interaction indicated that the effect of thermocycling was material-dependent, supporting an interaction between hydrothermal degradation and the physicochemical characteristics of each restorative material [14,23]. Only CAD/CAM-milled PMMA demonstrated a significant reduction in maximum fracture load after thermocycling, although it retained the highest fracture resistance under both aging conditions. This finding is consistent with previous studies reporting reduced load-bearing capacity of industrially polymerized CAD/CAM polymers following hydrothermal aging [2,25]. Water sorption may plasticize the polymer matrix by increasing intermolecular spacing and molecular mobility, thereby facilitating crack initiation and propagation [18]. Repeated thermal expansion and contraction may further generate internal stresses and promote microcrack formation [23,26]. Industrially prepolymerized PMMA materials are generally reported to exhibit relatively high degrees of conversion; if this characteristic also applied to the material investigated here, the limited potential for further post-polymerization could allow water-related degradation mechanisms to predominate during aging [18,25]. However, neither degree of conversion nor water sorption was measured in the present study, and this interpretation therefore remains speculative.

The additively manufactured photopolymer resin also showed no significant reduction in maximum fracture load after thermocycling. Recent studies further demonstrate that aging responses of additively manufactured interim resins are not uniform across materials and processing conditions. Alkhateeb et al. reported substantial effects of printing orientation and post-curing time on the fracture load of three-unit printed interim FDPs after thermocycling [20], whereas Almedarham et al. observed aging-related changes in flexural properties that depended on material modification and build direction [21]. More recently, Tavuz et al. demonstrated material-specific reductions in flexural strength following thermomechanical aging across multiple additively manufactured provisional resins, while some materials exhibited comparatively stable behavior [22]. Collectively, these findings support interpreting the absence of a significant reduction in maximum fracture load in the present photopolymer group as specific to the investigated resin–printer–processing combination and aging protocol rather than as evidence of general resistance of additively manufactured provisional materials to hydrothermal aging. However, aging significantly reduced the time to maximum load, indicating an alteration in load-deformation behavior despite the absence of a significant change in ultimate load-bearing capacity. Such behavior may reflect aging-related changes in the polymer network or interlayer interfaces [14,18]. Nevertheless, because time to maximum load at a constant crosshead speed reflects not only deformation of the restoration but also testing-system compliance and initial seating of the non-cemented restoration, this outcome should not be interpreted as a direct measure of material brittleness.

Fracture-location analysis revealed material-dependent failure patterns, with connector regions representing the predominant fracture sites across groups. This finding is consistent with previous studies identifying connectors as mechanically vulnerable regions of three-unit interim FDPs, where bending during occlusal loading generates tensile stress concentrations that may initiate crack propagation [2,12,26]. Because the mesial and distal connectors were standardized at 10 and 12 mm^2^, respectively, across all material groups, differences in fracture distribution may reflect the combined influence of material properties, connector geometry, and loading configuration.

Fracture location differed descriptively among the three materials. The additively manufactured photopolymer resin fractured predominantly at the mesial connector under both aging conditions, which may reflect the combined influence of the smaller mesial connector and the anisotropic characteristics of layer-by-layer fabrication [14,15]. Dentalon^®^ Plus showed a descriptive shift from predominantly mesial to distal connector fractures after thermocycling, whereas CAD/CAM-milled PMMA exhibited predominantly distal connector fractures irrespective of aging condition. These patterns suggest that failure location cannot be explained by connector dimensions alone but may reflect interactions among material properties, prosthesis geometry, and loading configuration [2,9,10]. Further fractographic or finite element analyses would be required to determine the mechanisms underlying these distributions.

Incomplete fracture was the predominant failure pattern across all restorative materials. Notably, no complete fractures occurred in the additively manufactured photopolymer resin group; however, this finding should be interpreted in conjunction with its substantially lower fracture resistance and should not be considered evidence of greater clinical durability. The lower load-bearing capacity of this material may have limited the strain energy accumulated before failure, thereby reducing the likelihood of catastrophic fracture [14]. Overall, fracture-pattern analysis provides complementary information to maximum fracture load by characterizing differences in failure behavior among restorative materials [2].

From a clinical perspective, the substantially higher fracture resistance of the investigated CAD/CAM-milled PMMA may favor its use when interim FDPs are expected to withstand prolonged or demanding functional conditions [2,5]. Nevertheless, its significant reduction in fracture resistance after thermocycling indicates susceptibility to hydrothermal degradation. Dentalon^®^ Plus exhibited intermediate and comparatively stable fracture resistance, whereas the investigated additively manufactured photopolymer resin showed the lowest load-bearing capacity [12]. These findings should be interpreted as specific to the materials and processing protocols evaluated rather than generalized to their respective fabrication technologies [16,26].

Several limitations should be considered when interpreting the present findings. First, the study evaluated only one commercially available additively manufactured photopolymer resin, one conventionally polymerized methacrylate-based resin, and one CAD/CAM-milled PMMA material. Moreover, because each fabrication workflow was represented by a different commercial material, material composition and manufacturing technique were not independently varied. Thus, the present experiment compares three specific material-fabrication workflow combinations and does not permit the independent effects of material chemistry and manufacturing technology to be separated. Therefore, the results are specific to the investigated materials and should not be generalized to all provisional restorative materials manufactured using similar fabrication workflows. Variations in resin formulation, polymer chemistry, filler content, and degree of conversion among commercially available products may result in substantially different mechanical behavior [5,24].

Second, fracture resistance was assessed under static compressive loading using a rigid, non-compliant titanium die model without cementation or simulation of periodontal ligament compliance. Although this configuration provided highly standardized support conditions, the absence of physiologic tooth mobility may have altered load transfer and stress distribution within the FDP, particularly at the connector regions, and may have resulted in higher fracture load estimates than those obtained using more compliant supporting models. Consequently, the recorded fracture loads should be interpreted as comparative laboratory values rather than direct estimates of clinical load-bearing capacity. In addition, the recorded time to maximum load reflected not only deformation of the restoration but also seating of the non-cemented restoration and compliance of the testing assembly. These experimental conditions therefore do not fully reproduce the complex biomechanical environment encountered clinically [14,26]. Because the investigated restorative systems likely differ in elastic modulus and overall structural compliance, the rigid support may not have influenced all groups to the same extent. A non-compliant substrate can modify connector bending and stress transfer in a material-dependent manner; therefore, the ranking observed under the present rigid-die conditions cannot be assumed to remain unchanged in a more compliant tooth-periodontal ligament model. Because elastic modulus was not measured in the present study, the magnitude or direction of this potential substrate–material interaction cannot be quantified. Furthermore, artificial aging in the present study was limited to thermocycling and did not incorporate cyclic mechanical loading or mastication simulation. Consequently, fatigue-related damage associated with repeated occlusal loading and its potential interaction with thermal stresses were not reproduced. Future studies combining thermocycling with mechanical cyclic loading before fracture testing would more closely approximate the combined thermal and mechanical challenges encountered during clinical service.

Third, the additively manufactured restorations were post-cured under ambient atmospheric conditions. Oxygen inhibition during post-polymerization may reduce the degree of conversion and cross-link density at the material surface, which is subjected to the highest tensile stresses during loading, thereby influencing fracture behavior [14,16]. Because the degree of conversion was not quantified, this explanation remains speculative, and the mechanical performance of the investigated photopolymer resin should be interpreted in the context of the specific post-processing protocol employed.

Finally, no spectroscopic assessment of degree of conversion, residual monomer analysis, water sorption or solubility testing, SEM-based fractography, dynamic mechanical analysis (DMA), differential scanning calorimetry (DSC), thermogravimetric analysis (TGA), or microstructural porosity analysis was performed. Consequently, the present study cannot directly characterize polymer-network organization, interfacial debonding, interlayer crack initiation, viscoelastic behavior, glass-transition characteristics, or thermal degradation of the investigated materials. Proposed explanations involving conversion, interlayer bonding, residual monomer content, porosity, water-induced plasticization, or continued post-polymerization should therefore be regarded as literature-supported hypotheses rather than experimentally verified mechanisms in the materials tested here [18,19]. Moreover, because the investigated photopolymer resin was processed on a printing platform for which it was not manufacturer-validated, the exposure parameters may not have been optimized for this specific formulation. The mechanical performance observed should therefore be considered specific to the resin–printer–post-curing combination evaluated. Because the same resin was not tested using a manufacturer-validated printing platform or resin-specific optimized exposure parameters, the direction and magnitude of any potential deviation from its performance under validated processing conditions cannot be determined from the present study.

Future studies should evaluate multiple commercially available materials within each fabrication workflow using standardized manufacturing and post-processing protocols. Degree of conversion should be assessed using FTIR or Raman spectroscopy, while SEM-based fractographic examination could help characterize fracture-surface features and potential interlayer or bulk failure patterns. Water sorption and solubility testing would further clarify hydrothermal effects, whereas micro-CT or other microstructural methods could quantify internal porosity and void distribution. Combining these analyses with fatigue-based mechanical testing would allow the proposed material-specific fracture and aging mechanisms to be evaluated directly rather than inferred solely from mechanical outcomes [25].

## 5. Conclusions

Within the limitations of this in vitro study, the three investigated material-fabrication workflow combinations differed significantly in maximum fracture load. CAD/CAM-milled PMMA exhibited the highest values, followed by the conventionally polymerized PEMA-based material, whereas the investigated additively manufactured photopolymer resin exhibited the lowest values. Under the present experimental conditions, thermocycling significantly reduced maximum fracture load only in the CAD/CAM-milled PMMA group. Because material chemistry and fabrication workflow were intrinsically coupled in the study design, their independent contributions cannot be separated. In addition, interpretation is limited by the rigid non-cemented testing model, the absence of cyclic mechanical fatigue, the non-validated resin–printer combination used for the photopolymer group, and the lack of direct physicochemical and fractographic characterization. Accordingly, the findings should be regarded as specific to the three investigated material-workflow combinations and experimental conditions rather than generalized to additive manufacturing, CAD/CAM milling, or conventional fabrication as technologies overall.

## Figures and Tables

**Table 1:** Distribution of experimental groups according to fabrication technique and aging condition.

Group	Material	Composition	Manufacturing Technique	Aging Condition	n
G1	Cyclone Temp Premium Resin	Methacrylate-based photopolymer resin	Additive manufacturing (LFS)	Non-aged	15
G2	Cyclone Temp Premium Resin	Methacrylate-based photopolymer resin	Additive manufacturing (LFS)	Artificially aged	15
G3	Dentalon^®^ Plus	Powder: polyethyl methacrylate (PEMA), polymethyl acrylate (PMA); liquid: n-butyl methacrylate, ethyl methacrylate, urethane acrylate	Conventional autopolymerization	Non-aged	15
G4	Dentalon^®^ Plus	Powder: polyethyl methacrylate (PEMA), polymethyl acrylate (PMA); liquid: n-butyl methacrylate, ethyl methacrylate, urethane acrylate	Conventional autopolymerization	Artificially aged	15
G5	Alliance PMMA Disc	Industrially prepolymerized PMMA	CAD/CAM milling	Non-aged	15
G6	Alliance PMMA Disc	Industrially prepolymerized PMMA	CAD/CAM milling	Artificially aged	15

LFS, low-force stereolithography; PMMA, poly(methyl methacrylate); PEMA, poly(ethyl methacrylate); PMA, polymethyl acrylate.

**Table 2:** Maximum fracture load (N) according to restorative material and artificial aging condition.

Aging Condition	AM Photopolymer Resin	Conventionally Polymerized Resin	CAD/CAM-Milled PMMA	Overall
Non-aged	177.71 ± 33.91 ^D^	460.33 ± 184.27 ^C^	1213.06 ± 126.65 ^A^	617.04 ± 459.95
Artificially aged	148.98 ± 34.81 ^D^	489.78 ± 163.80 ^C^	1050.83 ± 88.22 ^B^	563.20 ± 390.89
Overall	163.34 ± 36.79 ^c^	475.06 ± 171.96 ^b^	1131.95 ± 135.31 ^a^	590.12 ± 425.27
**Source of Variation**	***F***	***p***	**Partial η^2^ (95% CI)**	
Material	505.985	<0.001	0.923 (0.890–0.940)	
Artificial aging	4.499	0.037	0.051 (0.000–0.164)	
Material × artificial aging	4.997	0.009	0.106 (0.008–0.226)	

Values are presented as mean ± standard deviation. Data were analyzed using two-way ANOVA followed by Bonferroni-adjusted pairwise comparisons. Different lowercase superscript letters indicate significant differences among material main-effect means, whereas different uppercase superscript letters indicate significant differences among the six material × aging interaction groups (*p* < 0.05). Lowercase letters are assigned in descending order of the corresponding overall mean (a = highest). Effect sizes are expressed as partial eta squared (η^2^p) with 95% confidence intervals. AM, additively manufactured; PMMA, poly(methyl methacrylate).

**Table 3:** Time to maximum load (s) according to restorative material and aging condition.

Aging Condition	AM Photopolymer Resin	Conventionally Polymerized Resin	CAD/CAM-Milled PMMA	Overall
Non-aged	281.92 (179.25–321.30) ^AB^	264.56 (173.09–369.51) ^ABC^	243.99 (174.75–340.15) ^BC^	256.54 (173.09–369.51)
Artificially aged	233.01 (171.00–273.21) ^C^	274.87 (166.23–353.54) ^A^	266.74 (222.15–463.92) ^AB^	256.01 (166.23–463.92)
Overall	249.31 (171.00–321.30)	270.91 (166.23–369.51)	254.02 (174.75–463.92)	256.28 (166.23–463.92)
**Source of Variation**	***F***	***p***	**Partial η^2^ (95% CI)**	
Material	0.297	0.743	0.007 (0.000–0.058)	
Artificial aging	0.149	0.699	0.002 (0.000–0.057)	
Material × artificial aging	9.775	0.008	0.189 (0.051–0.319)	

Time to maximum load was recorded at a constant crosshead speed of 0.5 mm/min and therefore corresponds proportionally to crosshead displacement. Values are presented as median (minimum–maximum). Data were analyzed using two-way robust ANOVA followed by MCP2A robust multiple comparisons. Different uppercase superscript letters indicate statistically significant differences among interaction groups (*p* < 0.05). Effect sizes are reported as partial eta squared (η^2^p) with 95% confidence intervals. AM, additively manufactured; PMMA, poly(methyl methacrylate).

**Table 4:** Distribution of fracture locations.

Material	Aging Condition	Mesial Connector	Distal Connector	Pontic	Both Connectors
AM photopolymer resin	Non-aged	13 (86.7)	1 (6.7)	1 (6.7)	—
	Artificially aged	14 (93.3)	—	1 (6.7)	—
Conventionally polymerized resin	Non-aged	10 (66.7)	2 (13.3)	—	3 (20.0)
	Artificially aged	5 (33.3)	10 (66.7)	—	—
CAD/CAM-milled PMMA	Non-aged	5 (33.3)	6 (40.0)	—	4 (26.7)
	Artificially aged	4 (26.7)	9 (60.0)	—	2 (13.3)

Values are presented as n (%). *n* = 15 per group. AM, additively manufactured; PMMA, poly(methyl methacrylate).

**Table 5:** Distribution of fracture patterns.

Material	Aging Condition	Incomplete Fracture	Complete Fracture	Partial Fracture
AM photopolymer resin	Non-aged	15 (100)	—	—
	Artificially aged	14 (93.3)	—	1 (6.7)
Conventionally polymerized resin	Non-aged	5 (33.3)	10 (66.7)	—
	Artificially aged	11 (73.3)	1 (6.7)	3 (20.0)
CAD/CAM-milled PMMA	Non-aged	10 (66.7)	1 (6.7)	4 (26.7)
	Artificially aged	14 (93.3)	—	1 (6.7)

Values are presented as n (%). *n* = 15 per group. AM, additively manufactured; PMMA, poly(methyl methacrylate).

## Data Availability

The data supporting the findings of this study are available from the corresponding author upon reasonable request.

## Abbreviations

The following abbreviations are used in this manuscript:

AM	Additive manufacturing/additively manufactured
ANOVA	Analysis of variance
CAD/CAM	Computer-aided design/computer-aided manufacturing
FDP	Fixed dental prosthesis
LFS	Low-force stereolithography
PEMA	Poly(ethyl methacrylate)
PMA	Polymethyl acrylate
PMMA	Poly(methyl methacrylate)
PVS	Polyvinyl siloxane
SD	Standard deviation
STL	Stereolithography

## References

[1] Burns D.R., Beck D.A., Nelson S.K. (2003). A review of selected dental literature on contemporary provisional fixed prosthodontic treatment: Report of the Committee on Research in Fixed Prosthodontics of the Academy of Fixed Prosthodontics. J. Prosthet. Dent..

[2] Henderson J.Y., Korioth T.V.P., Tantbirojn D., Versluis A. (2022). Failure load of milled, 3D-printed, and conventional chairside-dispensed interim 3-unit fixed dental prostheses. J. Prosthet. Dent..

[3] Hahnel S., Krifka S., Behr M., Kolbeck C., Lang R., Rosentritt M. (2019). Performance of resin materials for temporary fixed denture prostheses. J. Oral Sci..

[4] Anusavice K.J., Shen C., Rawls H.R. (2012). Phillips’ Science of Dental Materials.

[5] Astudillo-Rubio D., Delgado-Gaete A., Bellot-Arcís C., Montiel-Company J.M., Pascual-Moscardó A., Almerich-Silla J.M. (2018). Mechanical properties of provisional dental materials: A systematic review and meta-analysis. PLoS ONE.

[6] Singh A., Garg S. (2016). Comparative evaluation of flexural strength of provisional crown and bridge materials—An in vitro study. J. Clin. Diagn. Res..

[7] Seredin P., Goloshchapov D., Kashkarov V., Ippolitov Y., Vongsvivut J. (2022). The Molecular and Mechanical Characteristics of Biomimetic Composite Dental Materials Composed of Nanocrystalline Hydroxyapatite and Light-Cured Adhesive. Biomimetics.

[8] Güth J.-F., Almeida e Silva J.S., Ramberger M., Beuer F., Edelhoff D. (2012). Treatment concept with CAD/CAM-fabricated high-density polymer temporary restorations. J. Esthet. Restor. Dent..

[9] Wiegand A., Stucki L., Hoffmann R., Attin T., Stawarczyk B. (2015). Repairability of CAD/CAM high-density PMMA- and composite-based polymers. Clin. Oral Investig..

[10] Alt V., Hannig M., Wöstmann B., Balkenhol M. (2011). Fracture strength of temporary fixed partial dentures: CAD/CAM versus directly fabricated restorations. Dent. Mater..

[11] Greuling A., Matthies A., Eisenburger M. (2023). Fracture load of 4-unit interim fixed partial dentures using 3D-printed and traditionally manufactured materials. J. Prosthet. Dent..

[12] Sadid-Zadeh R., Zirkel C., Makwoka S., Li R. (2021). Fracture strength of interim CAD/CAM and conventional partial fixed dental prostheses. J. Prosthodont..

[13] Mayer J., Stawarczyk B., Vogt K., Hickel R., Edelhoff D., Reymus M. (2021). Influence of cleaning methods after 3D printing on two-body wear and fracture load of resin-based temporary crown and bridge material. Clin. Oral Investig..

[14] Reymus M., Fabritius R., Keßler A., Hickel R., Edelhoff D., Stawarczyk B. (2020). Fracture load of 3D-printed fixed dental prostheses compared with milled and conventionally fabricated ones: The impact of resin material, build direction, post-curing, and artificial aging—An in vitro study. Clin. Oral Investig..

[15] Tahayeri A., Morgan M., Fugolin A.P., Bompolaki D., Athirasala A., Pfeifer C.S., Ferracane J.L., Bertassoni L.E. (2018). 3D printed versus conventionally cured provisional crown and bridge dental materials. Dent. Mater..

[16] Hwangbo N.-K., Nam N.-E., Choi J.-H., Kim J.-E. (2021). Effects of the washing time and washing solution on the biocompatibility and mechanical properties of 3D printed dental resin materials. Polymers.

[17] Legaz J., Sailer I., Mojon P., Lee H., Karasan D. (2023). Mechanical Properties of Additively Manufactured and Milled Interim 3-Unit Fixed Dental Prostheses. J. Prosthodont..

[18] Bettencourt A.F., Neves C.B., de Almeida M.S., Pinheiro L.M., e Oliveira S.A., Lopes L.P., Castro M.F. (2010). Biodegradation of acrylic based resins: A review. Dent. Mater..

[19] Schwantz J.K., Oliveira-Ogliari A., Meereis C.T.W., Leal F.B., Ogliari F.A., Moraes R.R. (2017). Characterization of bis-acryl composite resins for provisional restorations. Braz. Dent. J..

[20] Alkhateeb R.I., Algaoud H.S., Aldamanhori R.B., Alshubaili R.R., Alalawi H., Gad M.M. (2023). Fracture load of 3D-printed interim three-unit fixed dental prostheses: Impact of printing orientation and post-curing time. Polymers.

[21] Almedarham R.F., Al Dawood Z.H., Alatiyyah F.M., Akhtar S., Khan S.Q., Shetty A.C., Gad M.M. (2024). Flexural properties of additive manufactured resin designated for interim fixed dental prosthesis: Effect of nanoparticles, build direction, and artificial aging. Saudi Dent. J..

[22] Tavuz K.A., Al-Haj Husain N., Mätzener K.J., Ateş M.M., Eyüboğlu T.F., Özcan M. (2025). Evaluation of flexural strength of additively manufactured resin materials compared to auto-polymerized provisional resin with and without hydrothermal aging. J. Mech. Behav. Biomed. Mater..

[23] Gale M.S., Darvell B.W. (1999). Thermal cycling procedures for laboratory testing of dental restorations. J. Dent..

[24] Abdulmohsen B., Parker S., Braden M., Patel M.P. (2016). A study to investigate and compare the physicomechanical properties of experimental and commercial temporary crown and bridge materials. Dent. Mater..

[25] Stawarczyk B., Ender A., Trottmann A., Özcan M., Fischer J., Hämmerle C.H.F. (2012). Load-bearing capacity of CAD/CAM milled polymeric three-unit fixed dental prostheses: Effect of aging regimens. Clin. Oral Investig..

[26] Sokhal K., Kumar S., Aggarwal R., Kaur I., Thoidingjam B., Deokate A., Nandalur K.R., Nandalur K.R. (2025). Comparative evaluation of surface hardness of bis-acryl composite and polymethyl methacrylate-based provisional restorative materials: An in vitro study. Cureus.

